# Quantitative monitoring of the cytoplasmic release of NCp7 proteins from individual HIV-1 viral cores during the early steps of infection

**DOI:** 10.1038/s41598-018-37150-0

**Published:** 2019-01-30

**Authors:** Sarwat Zgheib, Iryna Lysova, Eleonore Réal, Oleksii Dukhno, Romain Vauchelles, Manuel Pires, Halina Anton, Yves Mély

**Affiliations:** Laboratory of Bioimaging and Pathologies, UMR 7021 CNRS, Faculty of Pharmacy, Université de Strasbourg, 67401 Illkirch, France

## Abstract

Fluorescence microscopy imaging of individual HIV-1 viruses necessitates a specific labeling of viral structures that minimally perturbs the infection process. Herein, we used HIV-1 pseudoviruses containing NCp7 fused to a tetracystein (TC) tag, labeled by a biarsenical fluorescein derivative (FlAsH) to quantitatively monitor the NCp7 protein concentration in the viral cores during the early stages of infection. Single particle imaging of individual pseudoviruses with defined ratios of TC-tagged to non tagged NCp7 proteins, together with theoretical modeling of energy transfer between FlAsH dyes, showed that the high packaging of TC-tagged proteins in the viral cores causes a strong fluorescence quenching of FlAsH and that the fluorescence intensity of individual viral complexes is an appropriate parameter to monitor changes in the amount of NCp7 molecules within the viral particles during infection. Interestingly, we observed a dramatic fluorescence increase of individual FlAsH-labeled pseudoviruses containing 100% TC-tagged NCp7 proteins in infected cells at 8 and 16 h post-infection. This effect was significantly lower for pseudoviruses expressing TC-tagged integrase. Therefore, this fluorescence increase is likely related to the cytoplasmic viral transformation and the release of NCp7 molecules from the viral complexes. This loss of quenching effect is largely reduced when reverse transcriptase is inhibited, showing that NCp7 release is connected to viral DNA synthesis. A spatial analysis further revealed that NCp7-TC release is more pronounced in the perinuclear space, where capsid disassembly is thought to be completed. Quantification of NCp7-TC content based on fluorescence quenching presented in this study evidences for the first time the cytoplasmic release of NCp7 during the remodeling of HIV-1 viral particles on their journey toward the nucleus. The developed approach can be applied to quantify dye concentrations in a wide range of nano-objects by fluorescence microscopy techniques.

## Introduction

During the early stages of HIV-1 infection, the viral core enters into the cytoplasm of the host cell and the reverse transcriptase initiates the synthesis of the viral DNA genome. As a result, the viral core undergoes a transformation from a ribonucleoprotein (RNP) complex into a reverse transcription complex (RTC) and then, finally into a preintegration complex (PIC). This transformation is thought to be associated with important structural rearrangements that involve a set of viral and cellular proteins. However, the precise timing and location of these conversions as well as the composition of the complexes are still debated^1–3^.

The initial RNP complex or ‘nucleocapsid’ comprises notably the Viral protein r (Vpr), integrase (IN), reverse transcriptase (RT) and the viral RNA dimer coated with more than 2000 molecules of NCp7 enclosed in a conical capsid structure composed of Capsid proteins^4^. According to a recently proposed model based on *“in-vitro”* experiments, the NCp7 molecules may be progressively released from the RTC during the synthesis of the viral DNA^5^. This hypothesis is based on the lower affinity of NCp7 for double stranded DNA as compared to the single stranded genomic RNA^6–8^, as well as on the probable dilution of the content of the capsid core due to its disassembly inducing a decrease of the RNP packaging. However, this release has not been evidenced so far *“in cellulo”*, in conditions mimicking a real infection.

To reach this objective, we used VSV-pseudotyped HIV-1 pseudoviruses (LVs) containing NCp7 or IN proteins fused to a TC tag, and expressing luciferase as a reporter of infection. The cellular entry of these LVs is different compared to WT HIV-1. The VSV glycoprotein on the surface of the pseudoviral particles enables their cell entry by endocytosis, independently of the presence of CD4 receptors on the cell plasma membrane. Once in the cytoplasm the viral cores escape the endosomes within several minutes and follow the infection course of the WT virus^9–11^. Therefore VSV pseudotyped HIV-1 are widely used as a model system for early stages of HIV-1 infection^12–16^. LVs were selectively labeled on the TC-tagged proteins by the arsenical fluorescein analogue FlAsH^17^. We hypothesized that the high concentration of NCp7-TC (~10 mM) in the RNP should cause strong self-quenching of the fluorescence of the FlAsH label. As a consequence, this quenching should decrease and thus the fluorescence of the particles should increase on release of NCp7-TC from the viral complexes into the cytoplasm. This hypothesis was validated by using pseudoviruses expressing various ratios of TC-tagged to non tagged NCp7 molecules, in order to mimic the increase in distance between NCp7 proteins in viral particles that is thought to result from progressive NCp7 release into the cytoplasm. By progressively increasing the percentage of tagged proteins from 3% to 100%, the fluorescence intensities measured on individual pseudoparticles were observed to first increase, then reach a maximum before gradually decreasing, leading to a very low fluorescence intensity when all NCp7 molecules have been tagged. These experimental data were found in good agreement with a theoretical model of FRET-based self-quenching of fluorophores.

In line with our expectations, the fluorescence intensity of individual FlAsH-labeled viral particles with 100% NCp7-TC or IN-TC in infected cells was observed to strongly increase between 2 and 16 h post infection with NCp7-TC, but not with IN-TC. The latter was used as a negative control because the amount of IN proteins bound to the genomic RNA or viral DNA is supposed to be constant during the trafficking of the viral particles in the cytoplasm^18^. These observations indicate a time-dependent release of NCp7 proteins into the cytoplasm. In order to quantify the fraction of NCp7-TC molecules released from the viral particles, we established a calibration curve by measuring directly in infected cells the fluorescence intensities of LVs containing different fractions of tagged NCp7 proteins. Based on this calibration curve, we could estimate that at 8 hours post infection approximately 40% of NCp7 molecules were released from the viral particles, while more than 70% were released at 16 hours of infection. Importantly, this increase of fluorescence with time was abolished in the presence of 3′-azido-3′-désoxythymidine (AZT), a specific inhibitor of reverse transcriptase, indicating that the release of NCp7 molecules is dependent on reverse transcription. Finally, the highest intensities of the viral complexes were observed in the perinuclear region, suggesting that NCp7 release mainly occurs prior to the nuclear entry. Overall, our data show for the first time the RT-dependent cytoplasmic release of NCp7 molecules during the early stages of HIV-1 infection.

## Results

### Virus design, production and infectivity assays

**Figure 1:**
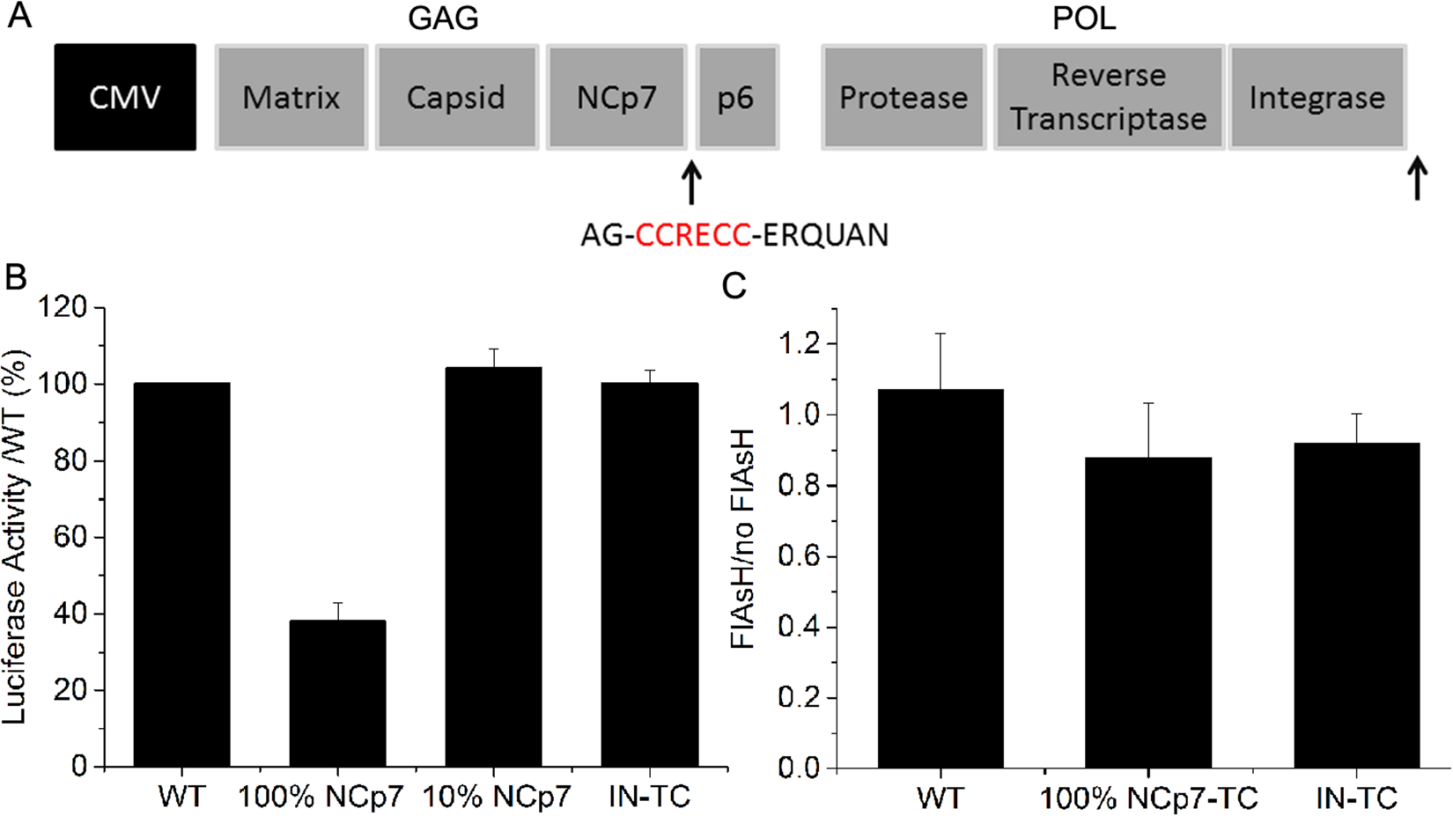
Effect of FlAsH-TC labeling on viral infectivity. (**A**) Scheme of packaging plasmids. The TC tag was inserted at the C-terminal end of NCp7 or Integrase. (**B**) Effect of the TC tag on pseudovirus infectivity. The luciferase activity in HeLa cells infected by pseudoviruses containing NCp7-TC or IN-TC relative to the activity of WT pseudoviruses is reported at 24 h post-infection. (**C**) Effect of FlAsH addition on luciferase activity in HeLa cells 24 h after infection with WT pseudoviruses and pseudoviruses containing 100% NCp7-TC or 100% IN-TC. Presented data correspond to ratio of luciferase activity in HeLa cells infected with FlAsH labeled LVs to the luciferase activity in HeLa cells infected with the LVs that underwent the same labelling procedure without FlAsH.

As NCp7 is a relatively small protein (7 kDa) endowed with highly conserved structure and key functions, its fusion to a tag may cause a loss of viral infectivity. Therefore, to produce a labeled pseudovirus with preserved infectivity, we used a small tetracystein (TC) tag (CCRECC) inserted at the C-terminal end of NCp7^19^ (Fig. 1A) in combination with FlAsH, a biarsenical fluorescein based label^17,20^. Besides its small size, this labeling system presents the key advantage that the fluorophore is added only after production of the pseudovirus, minimizing possible perturbations during the assembly process. The infectivity of the pseudoviruses, as measured by the luciferase activity on HeLa cells 24 h post infection, was fully preserved when 10% of NCp7 molecules were tagged or when the TC tag was inserted at the C-terminus of the IN (Fig. 1B). Even when 100% of the NC molecules were tagged by TC, the pseudoviruses remained infectious, showing a luciferase activity that still corresponds to 40% of the control pseudoviruses with no tagged proteins. This effect of the TC tag on the viral infectivity can be considered as moderate, knowing that numerous single point mutations in the highly conserved NC have been show to lead to a full loss of infectivity^21–23^. As the mature NCp7 protein and the NC domain of Gag exhibit multiple key roles in viral replication and as the effect of the TC tag is limited, it would be tedious to identify which steps of the viral lifecycle are impacted by the TC fusion.

The binding of FlAsH to NCp7-TC or IN-TC expressing pseudoviruses reduces their infectivity by only a small extent (Fig. 1C). In addition, FlAsH molecules do not affect the infectivity of WT pseudoviruses (Fig. 1C).

**Figure 2:**
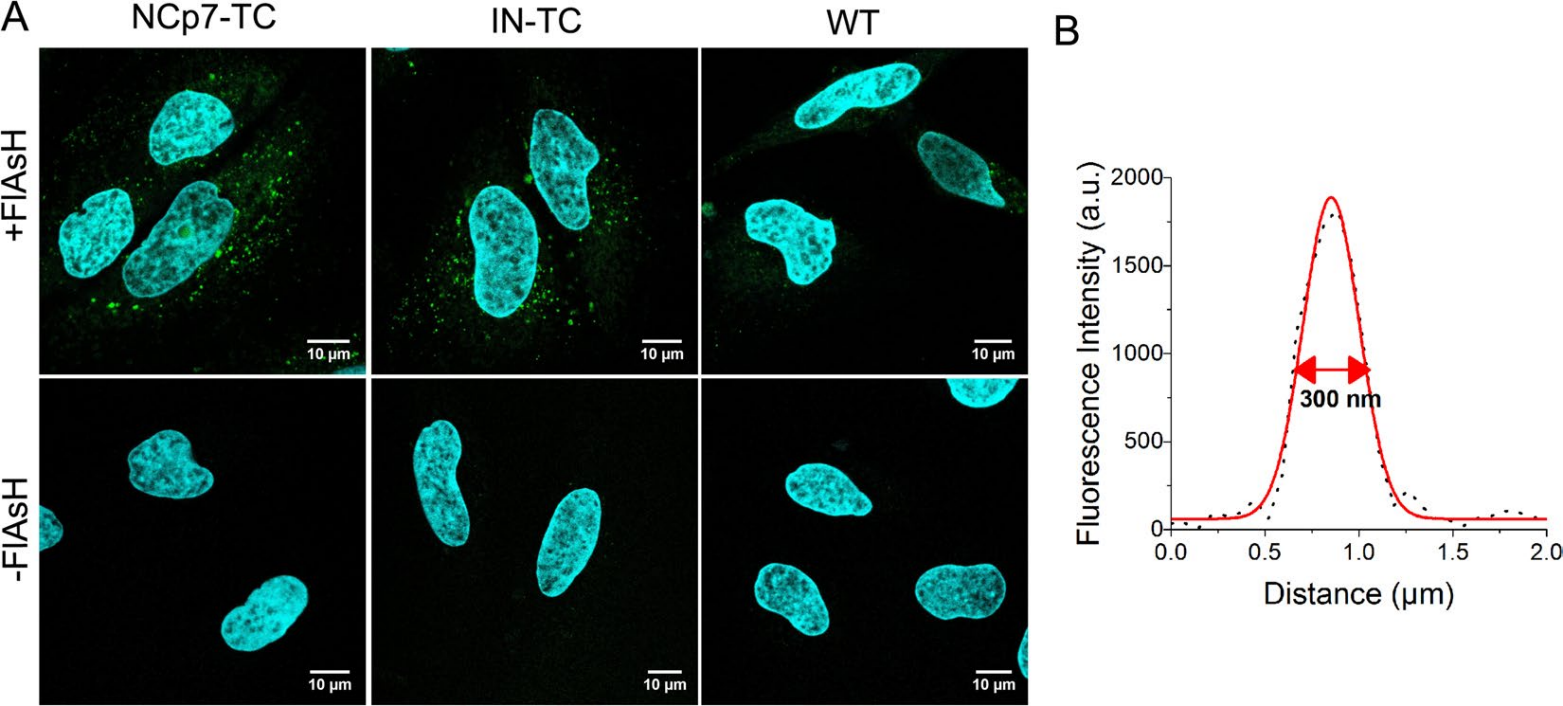
(**A**) Confocal images of HeLa cells infected with pseudoviruses (5 h.postinfection) containing NCp7-TC, IN-TC, or no TC tag in the presence (upper row) or in the absence (lower row) of FlAsH. The nuclei were stained by Hoechst 33258. (**B**) Intensity plot profile of a typical intracellular fluorescent spot fitted by a Gaussian function. Mean value of 100 measurements FWHM = 300 +/− 35 nm.

For imaging experiments, HeLa cells were incubated with labeled pseudoviruses during 2 h, then washed and incubated in complete medium for additional 6 to 14 h. The cells were fixed and imaged by confocal microscopy. Typical images are shown in Fig. 2A. Intense fluorescent spots are observed in the cytoplasm and sometimes in the nucleus of cells infected by viruses containing NCp7-TC or IN-TC (Fig. 2A upper line).

The VSV-g pseudotyped HIV-1 virus is a widely used model for the study of the early stages of HIV-1 infection^9–11^. Their VSV envelope glycoprotein ensures high tropism and rapid cell entry via endocytosis. The pseudoviral particles escape from the endo-lysosomes following an intravesicular pH decrease (reviewed in^24^), which usually occurs within 2 hours post infection^12,25–28^. In order to check whether this is also the case in our conditions, we monitored the co-localization of FlAsH labeled HIV-1 pseudoviruses with the lysosomal stain Lysotracker Deep Red and found that less than 14% of the pseudoviruses were still trapped in lysosomes at 2 hours post infection (Fig. S1 in the supplementary information).

Next, the intensity plot profiles of 100 intracellular fluorescent spots measured in 20 different infected cells were fitted by a Gaussian function. The average full width at half maximum (FWHM) value was 300 + −/35 nm (Fig. 2B), which is close to the theoretical diffraction limit of the microscope. Therefore, we estimate that 2 hours of contact is sufficient for the particles to escape from endosomes as free single viral particles.

Cells infected with wild-type pseudoviruses that do not contain any TC tag (WT) (Fig. 2A), show a residual labeling probably due to non-specific binding of FlAsH to the cysteine residues of viral proteins. Both the fluorescence intensity and density of the labeled particles in the images of the WT pseudoviruses are significantly lower as compared to the TC-containing pseudoviruses in Fig. 2A. These observations confirm the specificity of the FlAsH labeling. Since all samples were infected with the same quantity of p24, the low number of fluorescent spots in WT samples compared to TC-tagged samples implies that we detect only the population of the most intense WT particles. No fluorescent spot was detected in HeLa cells infected with viral particles that underwent the same labeling procedure but without adding the FlAsH fluorophore (Fig. 2A lower row). Moreover, the autofluorescence of non-infected cells was negligible in our experimental conditions.

Taken together, our data show that HIV-1 pseudoviruses containing NCp7-TC or IN-TC proteins labeled by FlAsH are infectious and can be imaged by fluorescence microscopy. These particles were further used as a model system for investigating the intracellular fate of NCp7 during the early stages of HIV-1 infection.

### Fluorescence quenching in FlAsH-labeled pseudoviruses expressing NCp7-TC

In mature HIV-1 virions, the RNA dimer is coated by more than 2000 molecules of NCp7^4^. The size of the capsid core being approximately 50–100 nm^29^, the local concentration of NCp7 in the HIV-1 virion is in the millimolar range. At such a high concentration, the emission of the fluorescein-based FlAsH fluorophores is thought to be strongly decreased, due to self-quenching effects^30^. Therefore, in order to evaluate the self-quenching of FlAsH in NCp7-TC-containing pseudoviruses, we measured the fluorescence intensities of FlAsH-labeled LVs containing various fractions of TC-tagged NCp7 and compared them to the values calculated from a theoretical model of FRET-based self-quenching of fluorophores^31^ (see Materials and Methods section for details).

**Figure 3:**
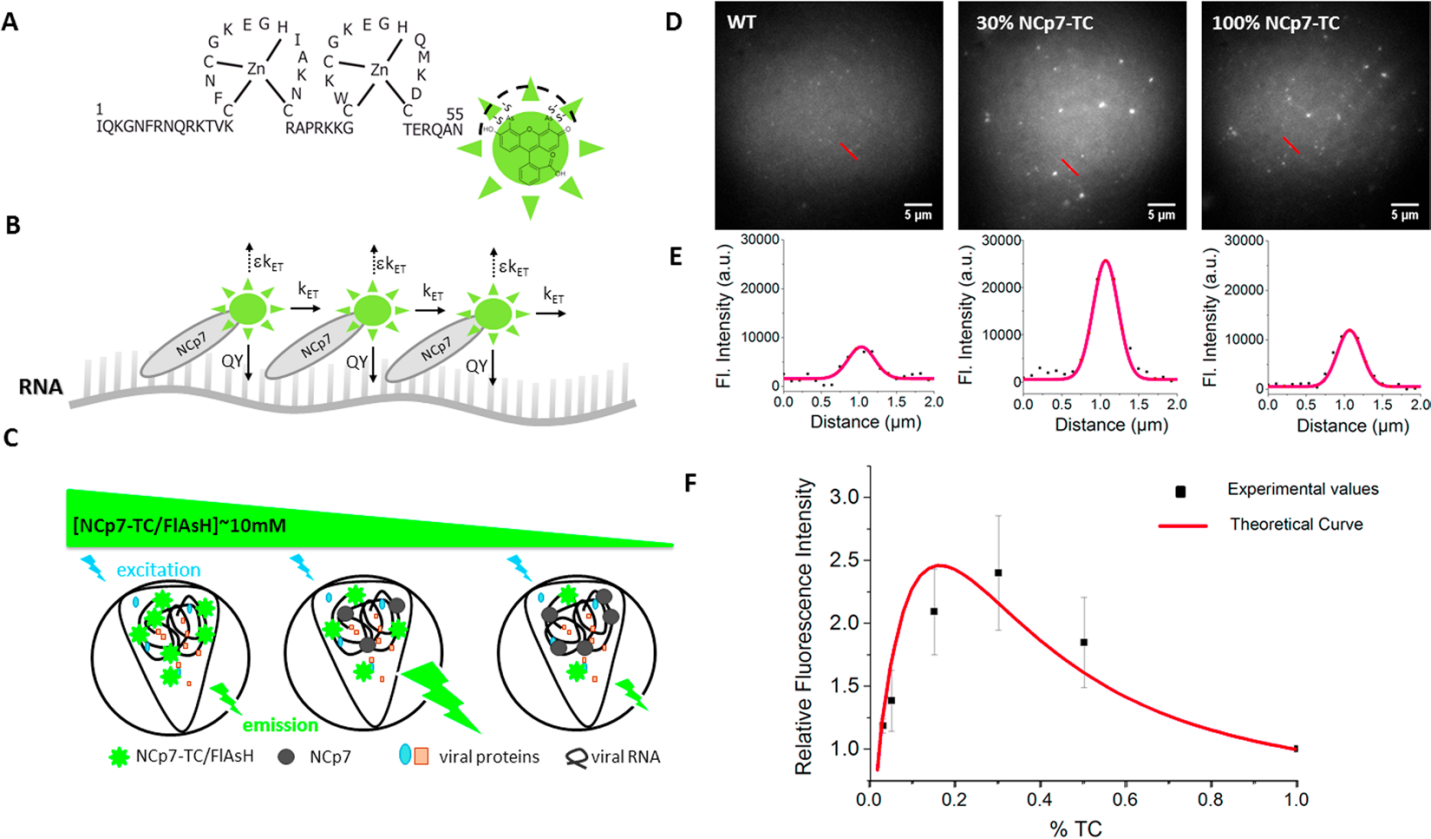
Evidence of fluorescence self-quenching in FlAsH-labeled pseudoviruses containing TC-tagged NCp7 proteins. (**A**) NCp7-TC protein with bound FlAsH. (**B**) FlAsH-labeled NCp7-TC molecules bound to RNA. The energy of FlAsH in its excited form is either emitted (QY), or lost non radiatively (εk_ET_) or transferred to one of its neighbors (k_ET_). (**C**) Principle of FlAsH quenching as a function of the fraction of NCp7-TC proteins. With increasing concentrations of FlAsH-labeled NCp7-TC proteins, the fluorescence emission first increases due to the increase in the concentration of emitters, and then decreases due to self-quenching effects that appear as a result of the close proximity of the emitters at high concentrations of FlAsH-labeled NCp7-TC proteins. (**D**) Typical fluorescence images of FlAsH-labeled pseudoviruses containing 0%, 30% and 100% of NCp7-TC protein (**E**) Intensity profiles of fluorescent spots (corresponding to the red lines in the fluorescence image) fitted by a Gaussian function. (**F**) Plot of the relative fluorescence intensity of pseudoviruses as a function of the percentage of TC-tagged NCp7 proteins. The intensity measured with particles containing a given fraction of NCp7-TC is expressed relative to the intensity of pseudoviruses containing 100% NCp7-TC. Squares represent the mean of three independent experiments with 600 particles measured and the red curve corresponds to the best fit to the theoretical model.

Theoretically, as shown in Fig. 3A,B, RNA bound NCp7-TC/FlAsH molecules can be represented as a network of fluorophores having a distance distribution H(r) in a confined volume V. When a fluorophore absorbs a photon, it is excited to an upper electronic state. Then, its relaxation can be realized either through the emission of a photon (with quantum yield QY), or a resonant energy transfer to the neighboring dyes (at a rate k_ET_), or a non radiative loss of energy due to packing-induced quenching by neighboring dyes (at a rate εk_ET_, with ε being a quenching factor). The total fluorescence emitted is dependent on the efficiency of quenching and the number of dyes in the viral core (equation 2, materials and methods section), which in turn is dependent on the concentration of NCp7 and the fraction of NCp7-TC in the viral core. Consequently, a decrease of the fraction of NCp7-TC in the viral core from 100% to lower values should cause an increase of the fluorescence emitted by the particle as shown in Figure 3C. At a certain point, however, the loss of intensity due to the decrease in the quantity of dyes in the viral core is expected to overcome the gain of intensity due to the decrease in quenching, and the intensity will thus drop. As a result, the dependence of the viral particle intensity on the quantity of dyes in the viral core will be nonlinear, with a single maximum.

In order to experimentally verify whether a concentration-dependent FlAsH quenching occurs in NCp7-TC-containing pseudoviruses, we replaced the packaging plasmid by a mix of packaging plasmids coding for TC-tagged and non-tagged NCp7 and produced pseudoparticles where the fraction of NCp7-TC was 0% 3%, 5%, 15%, 30%, 50%, and 100%. These pseudoparticles were then labeled by FlAsH dyes, deposited on fibronectin-coated coverglasses, fixed in 4% PFA and imaged by a wide-field microscope, equipped with an electron multiplying CCD that enables single photon detection. FlAsH-labeled WT pseudoviruses with non-tagged NCp7 proteins were used to quantify nonspecific labeling. Typical images are displayed in Fig. 3D. As shown on the intensity plot profiles in Fig. 3E, even the most intense WT pseudoviruses emitted a fluorescence signal about twice lower  compared to pseudoviruses with 100% TC tagged NCp7, which exhibit the strongest quenching effect. The intensity of 600 fluorescent spots was measured for each condition (see materials and methods section). The fluorescence intensity of non specifically labeled WT pseudoparticles was subtracted and the percentage of increase relative to viruses containing 100% NCp7-TC was plotted as a function of the TC fraction (Fig. 3F). The dependence was found to follow a single maximum curve, with a fluorescence emission increasing with the fraction of NCp7-TC up to 30%, and decreasing at higher fractions.

These experimental values were fitted to equation 2, which is derived from a theoretical model based on FRET-induced self-quenching of dyes, as detailed in Materials and Methods. Taking into account the probable heterogeneity of the pseudovirus population and variations of the FlAsH QY occurring during the labeling and fixation procedures, the experimental and theoretical data show a fairly good correspondence (the fitted values being within the standard error of the mean of 3 independent experiments). The floating fitting parameters *ε* and *V* that represent the quenching factor and the volume occupied by the NCp7-TC/FlAsH proteins bound to nucleic acids, respectively, were found to be 0.72 and 144000 nm^3^. Interestingly, the V value corresponds to ~70% of the predicted nucleocapsid volume, suggesting a partial compaction of the RNA/NCp7 complex. These results confirm the hypothesis of a concentration-dependent fluorescence quenching of FlAsH molecules inside the pseudoviral cores.

### Self-quenching of FlAsH fluorescence in infected cells

**Figure 4:**
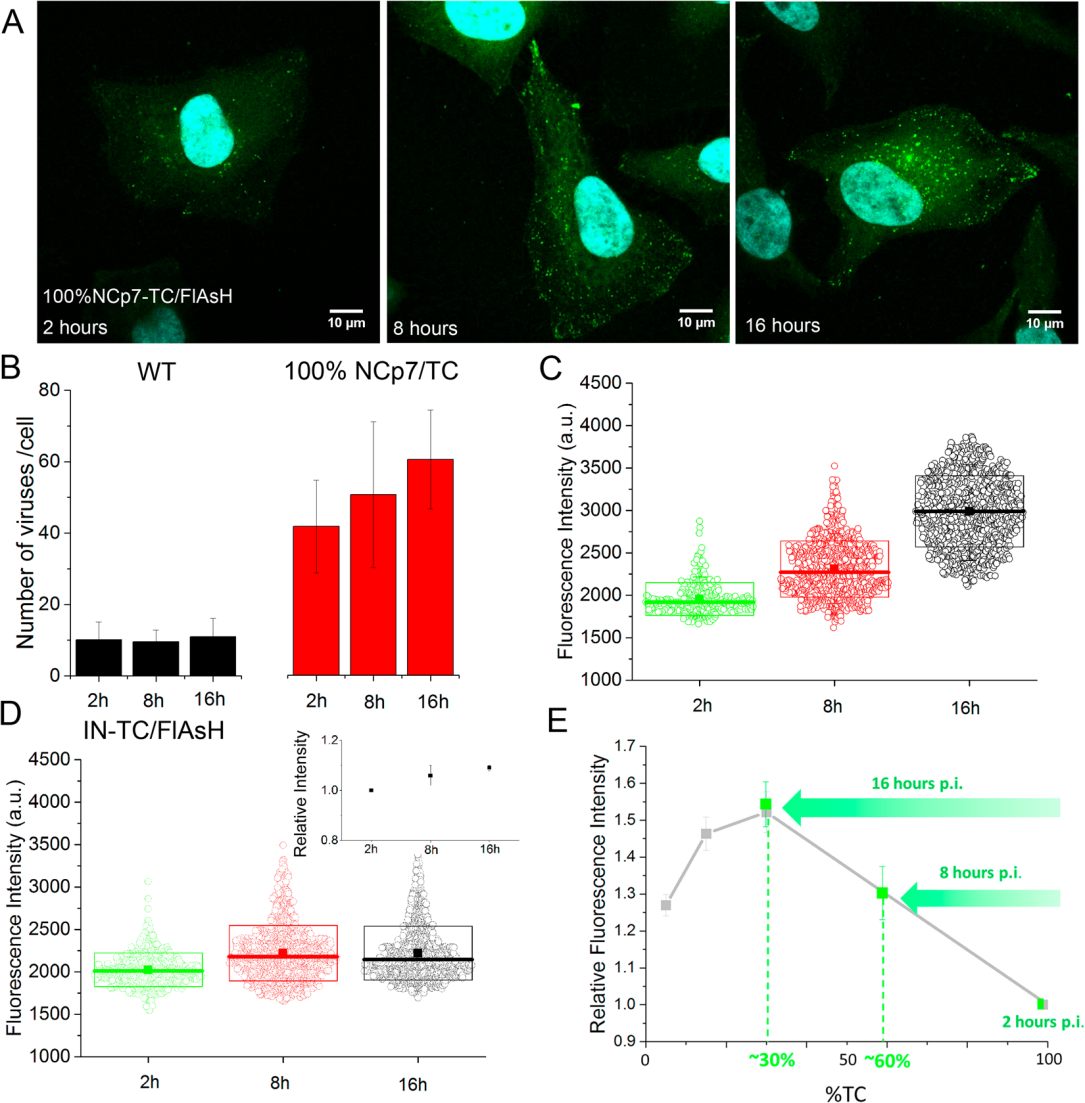
Self-quenching of FlAsH-labeled pseudoviruses in infected cells. Confocal images of cells infected with FlAsH-labelled pseudoviruses containing 15% (**A**) and 100% (**B**) of NCp7-TC. (**C**) Relative fluorescence intensity of pseudoviruses (squares) as a function of the percentage of NCp7-TC proteins. The mean fluorescence intensities of cytoplasmic spots in cells infected with pseudoviruses containing 5%, 15% and 30% of NCp7-TC were expressed as a ratio to the mean fluorescence intensity of cytoplasmic spots in cells infected with particles containing 100% of NCp7-TC proteins. 1000 fluorescent spots in 20–30 different cells were measured for each condition in three independent experiments.

In a next step, we checked whether the dependence of the fluorescence intensity of pseudoviral particles as a function of the fraction of NCp7-TC proteins is preserved during infection. Pseudoviruses containing 5%, 15%, 30% and 100% of TC-tagged NCp7 proteins were incubated with HeLa cells during 2 hours. After washing, the cells were fixed with 4% PFA and imaged by confocal microscopy. Optical sections in the middle of the cells were chosen for analysis (Fig. 4A,B).

The mean intensity of 1000 fluorescent spots in 20–30 different cells was measured for each condition and reported relative to the mean intensity of the same number of spots in cells infected by particles containing 100% NCp7-TC (Fig. 4C). As for free pseudoviruses, the dependence is nonlinear with a maximum for 30% of NCp7-TC. However, the absolute values of the fluorescence increase with TC percentage measured in intracellular conditions are somewhat lower than with isolated pseudoviruses. This is partly related to the difficulty to properly subtract the intensity of the FlAsH-labeled WT pseudoviruses that represent the level of non specific labeling. Indeed, the latter show high variability in their intensity as well as low S/N ratio due to cell autofluorescence and diffuse non specific FlAsH signal. Therefore the fluorescence intensity of the viral particles was simply corrected by subtracting the cell background. These data confirm that the self-quenching of FlAsH fluorescence is preserved in the intracellular environment during infection. Moreover, our data suggest that the NCp7-TC content of viral cores can be quantified during infection by measuring their fluorescence intensity and using the cellular data of Fig. 4C as a calibration curve.

### Time-dependent changes of NCp7-TC concentration in FlAsH-labeled pseudoviruses during infection

In order to monitor the possible concentration changes of FlAsH-labeled NCp7-TC molecules in pseudoviruses during the early steps of infection, HeLa cells were incubated with FlAsH-labeled pseudoviruses containing 100% NCp7-TC during 2 hours, 8 hours or 16 hours. After washing and fixation, the cells were stained with Hoechst and imaged by confocal microscopy.

**Figure 5:**
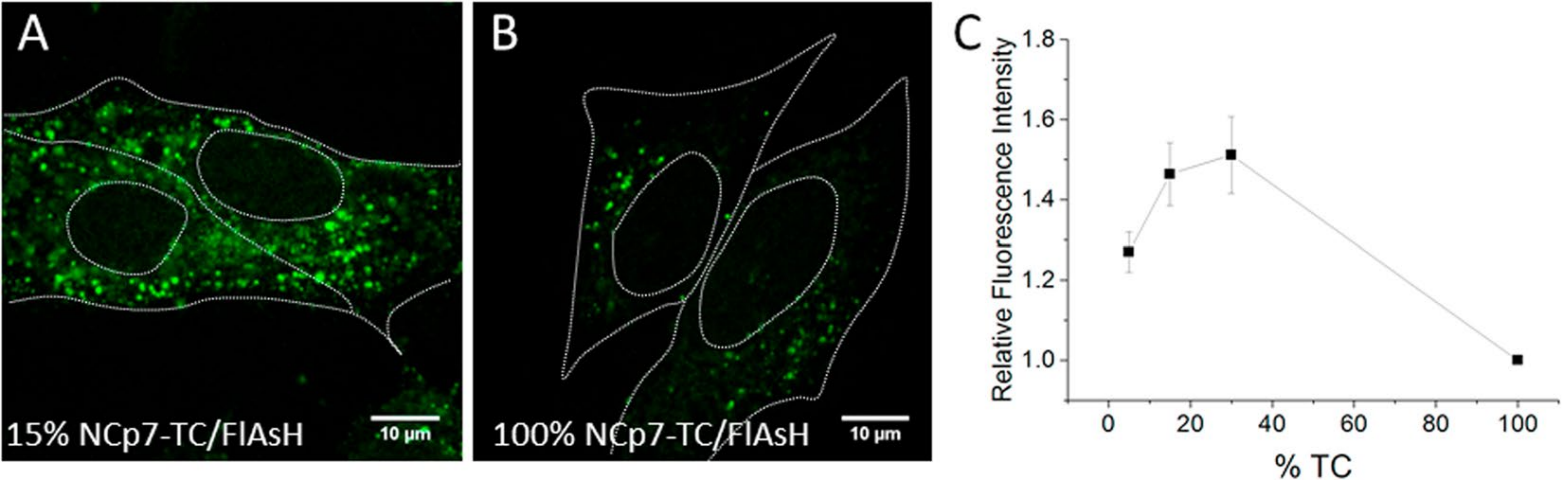
Time-dependence of the fluorescence intensity of FlAsH-labeled pseudoviruses containing 100% NCp7-TC during infection. (**A**) Typical confocal images of HeLa cells infected by FlAsH-labeled pseudoviruses containing 100% NCp7-TC, at 2, 8 and 16 hours post-infection. (**B**) Time dependence of the average number of particles detected per cell for FlAsH-labeled pseudoviruses containing WT NCp7 or 100% NCp7-TC. (**C,D**) Time dependence of the fluorescence intensity of 800–1500 fluorescent spots detected in 20 cells at each time point for pseudoviruses expressing (**C**) 100% NCp7-TC (mean fluorescence intensity values: 1900 +/− 200 at 2 h, 2300 +/− 300 at 8 h and 3000 +/− 400 at 16 h), (**D**) 100% IN-TC (mean fluorescence intensity values: 2000 +−/ 200 at 2 h, 2200 +/− 300 at 8 h and 2200 +/− 300 at 16 h). The boxes represent the SD, the thick solid lines represent the median and the squares represent the mean value. Insert: Time evolution of the fluorescence intensity of FlAsH-labeled pseudoviruses containing IN-TC relative to 2 hours p.i. Points represent the mean value of 3 independent experiments. (**E**) Evolution with time of the mean fluorescence intensity of FlAsH-labeled pseudoviruses containing 100% NCp7-TC. The mean fluorescence intensities at the various time points (green squares) were calculated from 4 independent experiments and were expressed as a ratio to the mean intensity value at 2 hours p.i. The grey squares and lines correspond to the calibration curve in Fig. 4C. Relative fluorescence increase of 100% NCp7-TC/FlAsH and IN-TC/FlAsH containing pseudoviruses were compared by 2-tailed Student’s t test. p = 0.04 at 8 h and p = 0.005 at 16 h.

Typical images (Fig. 5A) show intense fluorescent spots all over the cytoplasm and sometimes in the nucleus of infected cells. We next counted the number of viral particles detected in the infected cells (Fig. 5B) and measured their fluorescence intensity (Fig. 5C). While for control cells infected by FlAsH-labeled pseudoparticles containing non tagged NCp7, we could detect on average only 10 spots per cell independently on the incubation time (Fig. 5B), significantly higher numbers of spots were observed in cells infected with FlAsH-labeled pseudoviruses containing NCp7-TC. Moreover, the number of intracellular spots increased with time. Since similar observation was made for IN-TC containing viruses, we suppose that this increase likely corresponds to a progressive internalization of the pseudoviruses. It should be noted that the number of pseudoviruses detected per cell varied widely between individual cells in the same experiment and between different experiments. This could be caused by numerous factors such as the viral loss during the ultracentrifugation step, the cell shape, metabolic state and the phase of the cell cycle, as well as the heterogeneity of the viral deposit.

In parallel, a significant increase of the mean fluorescence intensity of FlAsH-labeled pseudoviruses containing 100% NCp7-TC was observed as a function of time (Fig. 5C). This increase is likely related to the loss of fluorescence quenching resulting from the decrease in the concentration of FlAsH-labeled NCp7-TC molecules in the viral core, due to their release into the cytoplasm. Since a small fraction of pseudoparticles may be trapped in late endo-lysosomes (Fig. S1) and since the fluorescence quantum yield of fluorescein-based dyes is highly sensitive to pH^32^, we checked the possibility that the observed fluorescence increase could be caused by the pH changes occurring in these compartments. To this end, the fluorescence spectra of FlAsH-labeled NCp7-TC containing pseudoviruses were measured at different pH values. As shown in the supplementary Fig. S2, the fluorescence emission of FlAsH dyes decreases by ~40% when the pH decreases from 7.2 to 4.8. Therefore, the observed increase in the fluorescence intensity in Fig. 5C is unlikely due to pseudoviruses trapped inside endosomes/lysosomes.

In support of our conclusion on the loss of fluorescence quenching, significantly lower evolution in fluorescence with time was observed with FlAsH-labeled pseudoviruses expressing IN-TC (Fig. 5D). IN-TC containing pseudoviruses were used as a negative control since only marginal variation of IN-TC proteins was anticipated, due to the key role of IN in the integration process. In addition its concentration in viral cores is 10-times lower compared to NCp7-TC, which should induce less FlAsH fluorescence quenching and therefore fewer fluorescence changes if part of IN-TC is released. The increase of NCp7-TC fluorescence at 8 and 16 hours was calculated relative to 2 hours p.i. At 8 hours post infection, the mean fluorescence intensity of the labeled pseudoviruses increased by 30 ± 11% and at 16 hours p.i. it reached 53 ± 10%. Based on the calibration curve obtained with FlAsH-labeled pseudoviruses containing various fractions of NCp7-TC (Figs 4C and 5E), we could roughly estimate that approximately 40% and 70% of the initially present NCp7-TC molecules were released from the viral complexes, after 8 and 16 hours of infection, respectively.

Taken together, a time-dependent increase of FlAsH fluorescence was observed with viral complexes containing NCp7-TC. This increase likely results from the gradual increase in the distance between FlAsH fluorophores in the viral complexes as a consequence of the release of FlAsH-labeled NCp7-TC molecules from the RTC.

### Dependence of FlAsH fluorescence quenching on the reverse transcription process

**Figure 6:**
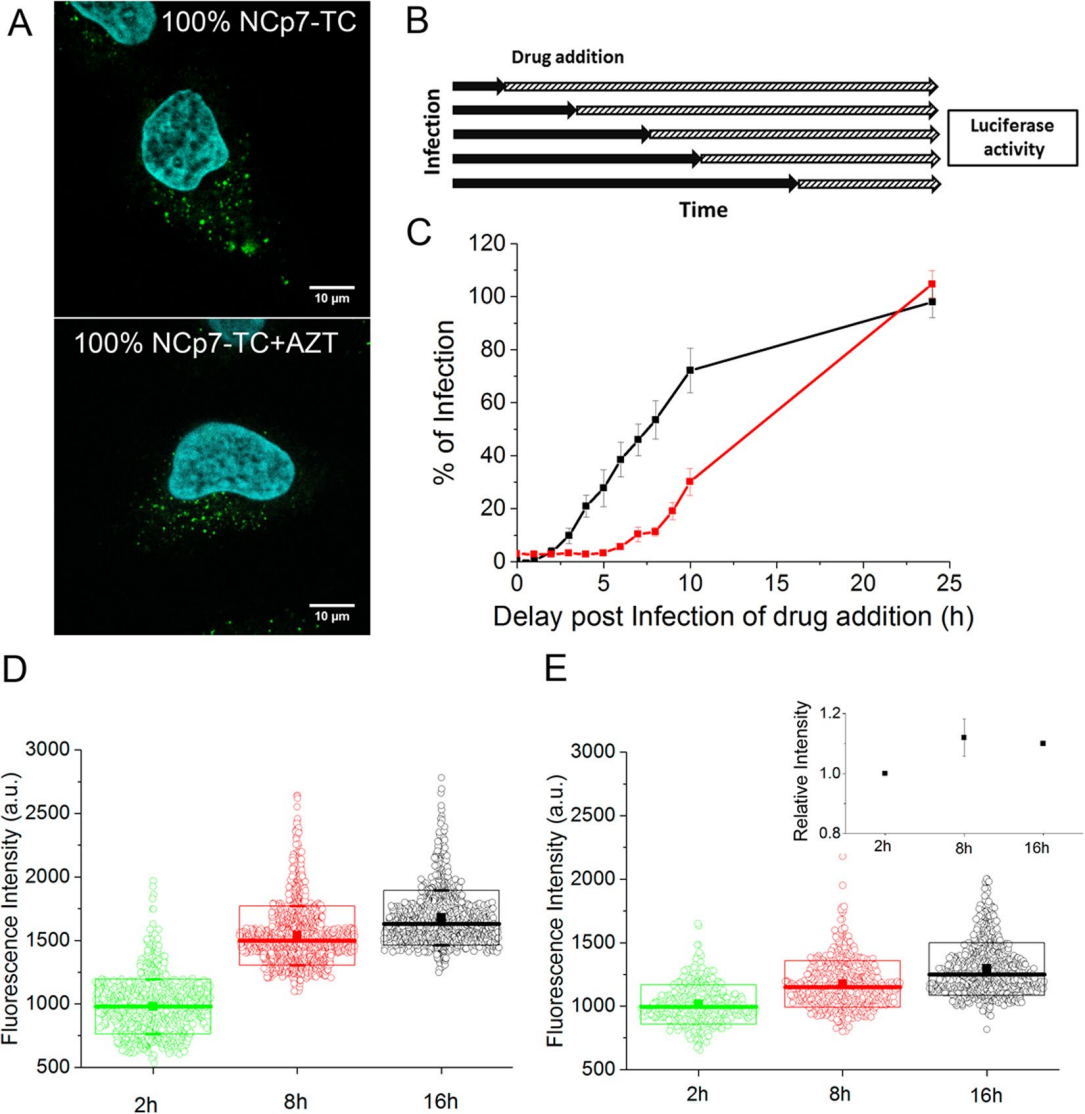
Effect of AZT on NCp7 release. (**A**) Confocal images of HeLa cells infected with FlAsH-labeled pseudoviruses containing 100% NCp7-TC in the absence or presence of 1 µM AZT (2 hrs p.i.). (**B**) Time of addition assay. After infection with viral pseudoparticles coding for luciferase, the cells were treated at different time points with 1 µM AZT or 2 µM Raltegravir. At 24 hours of infection, the cells were lysed and the infectivity was assessed by luciferase activity measurement. (**C**) Percentage of infection expressed as the luciferase activity relative to that of the untreated cells. Black curve correponds to AZT and red curve to Raltegravir treated cells. (**D,E**) Time evolution of the mean fluorescence intensity of ~1000 fluorescent spots detected in 20 cells at each time point in the absence or presence of AZT (**D**), mean fluorescence intensity values: 1000 +/− 150 at 2 h, 1400 +/− 200 at 8 h and 1700 +/− 200 at 16 h and in the presence of 1 µM AZT (E), mean fluorescence intensity values: 1000 +/− 150 at 2 h, 1150 +/− 200 at 8 h and 1300 +/− 200 at 16 h. Insert: Time evolution of the fluorescence intensity at given time points relative to 2 hours p.i. Squares represent the mean values of 3 independent experiments. Relative fluorescence increase of NCp7-TC/FlAsH in AZT free samples and AZT containing ones were compared by 2-tailed Student’s t test At 8 h p = 0.09 and at 16 h p = 0.007.

To determine whether the observed time-dependent fluorescence increase is related to the RTC to PIC conversion, we investigated the dependence of the FlAsH fluorescence changes on the reverse transcription process. As the release of NCp7 molecules is thought to be partly related to the conversion of the higher affinity single-stranded genomic RNA into the lower affinity viral DNA duplex^6–8^, inhibition of reverse transcription should partly prevent the release of NCp7 molecules. To perform this inhibition, we first investigated the timing of infection of our HIV-1 pseudoviruses. To this aim, we performed a time of addition assay on HeLa cells infected with HIV-1 pseudoviruses and treated at different time points (Fig. 6B) with specific inhibitors of reverse transcriptase (3’-azido-3’-deoxythymidine AZT) and integrase (Raltegravir). The measured luciferase activity expressed as a percentage of that of untreated cells revealed that AZT addition at 2 hours post infection reduces the luciferase expression by approximately 95%, indicating that reverse transcription was completed in only 5% of the cells (Fig. 6C). This percentage increased to 50% and 100% at 8 h and 16 h post-infection, respectively. For raltegravir, our data show that vDNA integration started at 6 h post infection and reached 50% at 12.5 h post-infection. Thus, the time-of-addition data confirmed that the post-infection time points of 2, 8 and 16 h are appropriate for evaluating processes that depend on reverse transcription.

In line with our expectations, comparison of the confocal images of infected cells in the absence and the presence of AZT, indicated that AZT did not significantly affect the intracellular distribution of the viral particles (Fig. 6A), but considerably limited the time-dependent fluorescence increase of the viral particles (compare Fig. 6D,E). Indeed, the fluorescence increase (mean value of 3 independent experiments) at 8 and 16 hours relative to 2 hours post infection was observed to not exceed 15% (Fig. 6E insert), indicating that the release of NCp7 molecules in relationship with reverse transcription is likely responsible for the fluorescence intensity changes of the viral particles.

### Spatial analysis of the intensity of FlAsH-labeled viral particles

**Figure 7:**
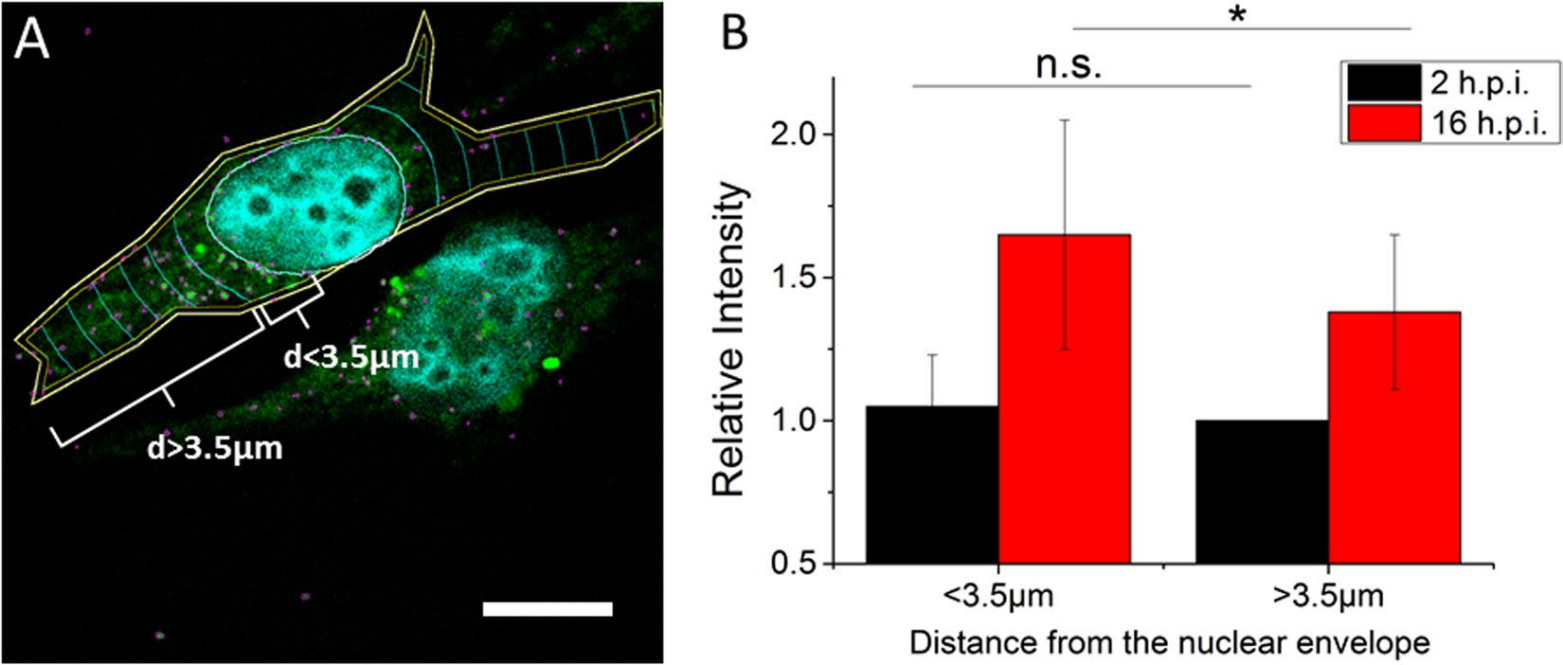
Spatial analysis of the fluorescent spots in FlAsH-labeled pseudoviruses. (**A**) Representative analyzed cell. A macro was used to divide the cell in concentric volumes. The distance between consecutive volumes was set to 3.5 µm. The nucleus is detected automatically through the Hoechst fluorescence. The contours of cells are drawn manually based on the cell autofluorescence. (**B**) Mean intensity of the fluorescent spots detected in the perinuclear region (<3.5 µm from the nuclear envelope) and in the rest of the cytoplasm (>3.5 µm from the nuclear envelope). The fluorescence intensity values in the regions distant by more than 3.5 µm from the nucleus at 2 h post infection were taken as 100%. The relative increase in the perinuclear region at 2 h is negligible, being 5 ± 18%. At 16 hours post-infection, the fluorescence intensity of individual pseudoviruses increases by 37 ± 20% in the cytoplasm and 65 ± 40% close to the nucleus. The data were compared by 2 tailed student test p < 0.04, Plotted values represent the mean ± SD of ~50 spots/cell and N = 20 cells for each condition.

Following cell entry, the pseudoviruses are transported from the plasma membrane towards the nucleus via the microtubule network^33,34^. It has been shown that reverse transcription and uncoating processes are related to this transport^27,35^. Therefore, to verify if the release of NCp7-TC/FlAsH is more pronounced in the perinuclear space, we compared the intensities of the viral pseudoparticles located in the “perinuclear region” (less than 3.5 µm from the nuclear envelope) to those located further away in the cytoplasm. To this aim, we analyzed the dependence of the fluorescence intensity of FlAsH-labeled pseudoviruses containing 100% NCp7-TC at 2 and 16 h post-infection as a function of their localization relative to the nuclear envelope using a home-made Image J macro (Fig. 7A). This macro created concentric areas with adjustable width around the nucleus and provided the mean intensity of the fluorescent spots in each area. Though a 3D analysis would be more suited for this analysis, a 2D analysis of a plane at the middle of the cell is appropriate for our comparison, if we assume a random diffusion of the pseudoviruses in and out of the focal plane.

At 2 h post infection, the intensity of the spots was found to be homogeneous all over the cytoplasm. At 16 h post infection, the fluorescence intensity of the pseudoviruses significantly increases for the particles closer to the nucleus, suggesting that the viral complexes in the vicinity of the nucleus contain less NCp7-TC/FlAsH molecules (Fig. 7B). However, no diffuse fluorescence corresponding to the accumulation of the released NCp7-TC/FlAsH molecules could be evidenced in the perinuclear region. This is likely the consequence of a stepwise and time-dependent release of NCp7 molecules, which prevents a sufficiently high local concentration of NCp7 molecules from being attained to be observable. This local concentration may be further diluted by the fast diffusion of the FlAsH-labeled NCp7-TC proteins in the cytoplasm and even in the nucleus^36^, their photobleaching and their proteolytic degradation.

## Discussion

Fluorescence microscopy imaging of individual HIV-1 viruses requires a labeling of viral structures that should not perturb the course of infection. Among the different labeling strategies, the tetracystein/FlAsH approach is of particular interest, due to the small size of the tag. Therefore in order to investigate the intracellular fate of HIV-1 NCp7 proteins in a context mimicking the real infection, we have produced HIV-1 pseudoviruses containing NCp7 proteins with a small tetracystein tag fused to their C-terminus. Importantly, these pseudoviruses showed only a limited decrease in infectivity as compared to pseudoviruses containing non tagged NCp7 proteins, indicating that the tag is small enough to preserve the activity of this highly conserved protein endowed with key functions all over the viral life cycle. After binding of the FlAsH dyes to the TC tags, the pseudoviruses containing NCp7-TC proteins were imaged by fluorescence microscopy. The size of HIV-1 viral cores being approximately 100 nm^29^, the particles were perceived in the cytoplasm of infected HeLa cells as clearly visible fluorescent spots of size close to the diffraction limit.

By measuring the fluorescence intensity of individual pseudoparticles that contained different proportions of tagged and non-tagged NCp7, we evidenced a quenching of FlAsH fluorescence in particles containing more than 30% of NCp7-TC. This was expected because fluorescein-based molecules have been described to undergo self-quenching at millimolar concentration^30^. Fluorescence quenching being highly dependent on the local dye concentration, this property is used to monitor various processes such as the release of encapsulated molecules from liposomes, membrane permeation or changes in water volume in lipid vesicles and cells^37–40^. In most of these studies, the fluorescence increase due to the loss of quenching was monitored by spectroscopy measurements, but in this work it was measured on the single particle level by fluorescence microscopy. In order to confirm the quenching of FlAsH fluorescence in HIV-1 viral cores, our experimental data were compared to a theoretical model of FRET-based self-quenching^31^. Despite the probable heterogeneity of the population of pseudoviruses in each condition, the experimental data could be well fitted with the theoretical model, validating the use of FlAsH fluorescence to follow NCp7-TC concentration changes in HIV-1 pseudoviruses during infection.

Next, by monitoring individual FlAsH-labeled pseudoviruses containing 100% NCp7-TC in infected cells at 2, 8 and 16 hours post infection, we evidenced a significant increase in their fluorescence intensity with time. This increase was not caused by any cellular or environmental modification as the fluorescence changes were significantly lower for pseudoviruses containing IN-TC. Moreover, this increase was largely abolished by AZT, a specific inhibitor of reverse transcriptase. These data strongly suggest a decrease of the concentration of FlAsH-labeled NCp7-TC in the viral cores during viral DNA synthesis.

Data from the literature^12,15,41^ and our data (Figs 5 and 6) indicate that 2 hours of cell/pseudovirus incubation is enough to ensure efficient infection and avoid the onset of reverse transcription in most of the virions. Therefore, this time point was used as a reference time in our experiments. As reverse transcription was negligible during the two first hours of incubation, no significant difference of intensity was observed when the cells were incubated with the pseudoviruses with or without AZT. Later on, at 8 and 16 hours p.i. the fluorescence intensity increased in the AZT-free samples. The fluorescence increase was significantly lower in the presence of AZT, indicating that the release of NCp7-TC/FlAsH is related to vDNA synthesis.

These observations are in line with a previous model describing a release of NCp7 molecules during the RTC to PIC conversion. Indeed, transcription of ssRNA into dsDNA is thought to cause a dissociation of an important fraction of NCp7 molecules from RTC complexes^5^, due to the lower affinity of NCp7 for dsDNA as compared to ssRNA^6–8^. Several studies have highlighted the relationship between the biological role of NCp7 molecules and their density on RNA. The initial high number of NCp7 molecules on the viral RNA dimer enabling its full coating is thought to play an essential protective role inhibiting an early onset of reverse transcription^42^. Then, when the NCp7:nucleotide ratio decreases, the protein role likely switches from genome protection to reverse transcription chaperoning^6^. In agreement with this hypothesis, our data evidence a release of NCp7 proteins from the viral cores during first 8 and 16 h of infection, when reverse transcription is thought to take place (our data and^41^). Our results indicate that at 8 hours post infection, when according to the time of addition assay ~50% of viral DNA is thought to be synthetized (~70% according to^41^), roughly 40% of NCp7 molecules are released. Then between 8 and 16 hours post infection, when the pace of DNA synthesis slows down, the NCp7-TC release continues and reaches ~70% at 16 hours post infection. Since reverse transcription is mostly completed in this time interval, this increase may also reflect other steps of viral core transformation, most probably the uncoating process.

Indeed, the release of NCp7–TC during RTC remodeling might also be favored by the disassembly of the viral capsid that should dilute its content and facilitate the dissociation of NCp7 molecules bound to the viral nucleic acids. Several reports have shown a close relationship between reverse transcription and capsid disassembly, since inhibition of reverse transcriptase delays uncoating^14,43,44^. Therefore we assume that the inhibition of RT by AZT prevents the initiation of the uncoating process, which may further block the release of NCp7 molecules from the viral complexes.

Though capsid disassembly has been clearly connected to reverse transcription^14,26,43,44^, its spatio-temporal aspects remain elusive. Based on the protective role of the capsid core for the viral genome^45–47^ together with its role in the nuclear import of the PIC^48–53^ and the presence of capsid proteins in the nucleus^28,54^, a model of “late” uncoating was suggested according to which the conical capsid core remains complete until the virus docks to the nuclear envelope^1,55–57^. On the other hand, several immunofluorescence imaging studies indicate that the capsid cone loses its integrity earlier, during the cytoplasmic transportation towards the nucleus, before completion of reverse transcription^14,58^. In our study, the analysis of the fluorescence intensity of FlAsH-labeled pseudoparticles as a function of the distance from the nucleus indicated that NCp7 release is more efficient in the vicinity of the nucleus. This accentuated release of NCp7 in the proximity of the nuclear envelope also suggests a close relationship between the maturation of the viral complexes and their transport towards the nucleus, in line with recent reports showing that microtubule disruption or knock down of dynein delays the uncoating process^27,35,59^.

In conclusion, a fluorescence quenching effect is used in this single virus imaging study to quantify the cytoplasmic release of NCp7 molecules from HIV-1 pseudoviral complexes during their journey to the nucleus. This release increases with time and is inhibited by AZT, indicating that NCp7 molecules are released during and/or after vDNA synthesis. Our data further indicate that NCp7 molecules are preferentially released close to the nucleus, where the capsid disassembly should be completed. Besides giving fundamental information, the imaging approach that we developed might be useful to clarify the mechanism of action of specific NCp7 inhibitors and inspire the design of new therapeutic strategies. Moreover, the presented experimental approach can be applied to quantify the dye concentration in a wide range of nano-objects by fluorescence microscopy techniques.

## Materials and Methods

### Cell culture

HeLa and 293T cells were cultured in Dulbecco’s modified Eagle medium supplemented with 10% fetal bovine serum (Invitrogen Corporation, France) at 37 °C in a 5% CO_2_ atmosphere.

### Production of vectors and viruses

Plasmids were cloned according to Perreira *et al*.^19^. The tetracystein tag (CCRECC) was added to the C-terminal of Integrase or NCp7 protein.

HIV-1 pseudotyped viruses were prepared following a protocol based on Arhel *et al*. 2006 and Lelek *et al*.^15,33^. 293T cells were co-transfected by the VSV-G coding plasmid pMD2-G, the packaging plasmid pCMV_dR8.91 and the transfer vector pSicor-luciferase using JetPEI (PolyPlus transfection, France) according to supplier’s recommendations. 24 h post transfection, the culture medium was replaced by serum free medium. The cell supernatant collected 48 h post transfection was filtered through 0.45 µm low binding filters (Millipore, France) to eliminate large cellular debris and was further concentrated by centrifugation in Vivaspin collection tubes with a cutoff of 50 kDa (Sartorius, Intec, France). The concentration of p24 antigen was measured by a p24 ELISA test (Innotest HIV Antigen mAb, InnoGenetics, Belgium). The aliquots of viral particles were stored at −80 °C.

The infectivity of the pseudoviruses was measured by luciferase luminescence 24 h after infection. HeLa cells were plated in triplicate for each type of pseudovirus in 96-well dishes and infected with equivalent amounts (0.5–11 ng) of p24 viral antigen mixed with 8 µg/mL polybrene solution in DMEM. After 24 h, the cells were washed with PBS and lysed using a cell culture lysis buffer (Promega, France) supplemented with 0.5% Triton-X100 during 20 min at room temperature. Luciferase activity was measured with a luciferase assay (Promega, France) according to supplier’s recommendations and a Berthold TriStar luminometer (LB941, Berthold Technologies, Germany).

Particles with different proportions of TC-tagged and WT NCp7 proteins were obtained by transfecting 293 T cells with mixtures of plasmid vectors encoding both proteins.

For the time of addition assay, HeLa cells were seeded in 96-well plates (5.10^3^ cells per well). On the next day, the cells were infected with HIV-1 pseudoviruses (1.7 ng of p24 eq.) with 8 µg/mL of polybren-containing medium. Between 1 and 10 h post-infection, AZT (1 µM final concentration), Raltegravir (2 µM final concentration) or DMSO (control samples) were added every hour in four wells. Finally, at 24 h post-infection the luciferase activity was measured and the percentage of infection was calculated for each time point as the ratio of the luciferase activity relative to control samples. The results represent the mean values of four independent experiments performed in quadruplicate.

### FlAsH labeling of the pseudoviruses

The viral supernatants (containing 0.5–1 µg of p24) were labeled at room temperature with 0.8 µM FlAsH-EDT2 (Molecular Probes, France), 1 µM beta-mercaptoethanol, 1 µM Tris (2 carboxyethyl) phosphine (TCEP) and 10 µM 1,2-ethanedithiol in a total volume of 1 mL of serum free medium. All chemicals were purchased by Sigma Aldrich, (St. Louis, Missouri, USA) or by Alpha Aesar-Thermo Fisher Scientific (Karlsruhe, Germany). The unbound fluorophores were washed out by an ultracentrifugation step (100,000 g during 30 min at 4 °C) and virus-containing pellets were resuspended in 200 µL of PBS and deposited on HeLa cells cultured on poly-L-lysine coated cover glasses, or in Ibi-Treat 4 well chambers (μ-Dish IBIDI, Germany). Typically, an equivalent of 50–100 ng of p24 protein was deposited on 50000 cells. After 2 h of infection at 37 °C, the cells were washed in PBS and incubated for additional 6–14 h at 37 °C. The samples were stained by 1 μM Hoechst 33342 (Molecular Probes, France) during 10 min and fixed with 4% PFA during 10 min at room temperature. For co-localization experiments, HeLa cells were infected with FlAsH-labeled 100% NCp7-TC or IN-TC containing pseudoviruses during 2 hours in PBS. For the last 30 minutes of infection, the PBS was replaced by complete medium containing 50 nM LysoTracker Deep Red (Thermo Fisher Scientific, France). The samples were fixed during 15 minutes with 2% PFA.

For imaging cell-free isolated particles, labeled pseudoviruses were resuspended in PBS after the ultracentrifugation step and deposited on poly-L-lysine- or fibronectin-coated glass cover slips. After 30 min, the samples were fixed with 4% PFA.

For spectroscopic measurements, the labeled pseudoviruses were resuspended in 4% PFA and diluted 10-fold in 10 mM phosphate or citrate/phosphate buffer containing 140 mM NaCl at different pH.

### Fluorescence Spectroscopy

The fluorescence emission of NCp7-TC/FlAsH labeled pseudoviruses was measured using a FluoroMax4 spectrophotometer (Jobin-Yvon, France). The samples were excited at 480 nm and the emission spectra were corrected for the emission of the buffer and lamp fluctuations.

### Fluorescence Microscopy

Wide-field microscopy was performed on a home-built setup based on a Nikon Eclipse Ti inverted microscope with a high-numerical aperture (NA) TIRF objective (Apo TIRF 100×, oil, NA 1.49, Nikon). Samples were illuminated with a 488 nm laser diode (15 W cm^−2^, Spectra Physics). The fluorescence emission was detected on an electron multiplying CCD digital camera (ImagEM, HAMAMATSU) with a pixel size of 106 nm and an exposure time of 100 ms. A StopLine Notch filter 488 nm (Semrock) and a 488 nm long pass filter (Semrock) were used to filter the FlAsH fluorescence signal.

Confocal microscopy experiments were performed on a Leica SPE microscope equipped with a 63 × oil immersion objective (HXL PL APO CS, 63 × oil, NA 1.4, Leica). Hoechst, FlAsH and Lyso Tracker Deep Red were excited with 405 nm, 488 nm and 632 nm lasers, respectively. The emitted fluorescence was detected by a PMT detector in a spectral range of 415–450 nm for Hoechst, 500–600 nm for FlAsH and 650-750 nm for LysoTracker Deep Red. The fluorescence crosstalk between the signals was carefully checked prior the experiments.

### Image processing and analysis

Image processing and analysis were carried out using a ImageJ software^60^. To quantify the fluorescence intensity of the FlAsH-labeled pseudoviruses deposited on the cover slip, the plot profiles of the fluorescence spots were fitted with a Gaussian function. The spots with a FWHM >400 nm were rejected, and only the fluorescence intensities of the spots corresponding to well-focused single particles were used for quantification. For each proportion of TC-tagged NC to non-tagged NCp7 proteins in the pseudoviruses, the mean intensity of 200 to 300 viral particles was calculated and subtracted from the mean value of the FlAsH-labeled WT pseudoviruses containing non-tagged NCp7 proteins. These intensities were then reported as a ratio to the fluorescence intensity of FlAsH-labelled pseudoviruses containing 100% TC-tagged proteins. For each condition, three independent experiments were performed.

In order to measure the fluorescence intensity of the viral particles in infected cells, the images were first thresholded to isolate the fluorescent spots corresponding to these particles. Then, a particle analysis function was used to identify their borders. Their coordinates were saved in ROI manager and applied to the initial image to measure the mean fluorescence intensity of the spots. Since the signal of WT virus containing non tagged NCp7 was almost negligible, the cellular background was used for “baseline” subtraction. In each experiment, the measurements were realized on 15–20 images per condition and three independent experiments were performed to confirm the results.

The spatial distribution of the intensities of the fluorescent spots was analyzed by using a home-built macro for ImageJ software^60^. In a first step, the images of the blue and green channels representing the nucleus (DNA labeled by Hoechst) and the FlAsH-labelled viral particles, respectively were separated. The autofluorescence signal of the green channel was used to generate a mask of the cell contours and the shape of the nucleus was detected automatically on the binary image after thresholding the Hoechst channel. The macro generated then automatically concentric regions around the nucleus and detected the number and mean intensity of the green particles in each region. The threshold values and the thickness of the concentric crowns were setup up by the user.

The co-localization of FlAsH labeled pseudoviruses with the lysosomes was analyzed by an object based approach using the JACoP Pluggin for ImageJ^61^. Confocal stacks were first thresholded in order to detect the spots in both channels. Next, since both the pseudoviruses and the lysosomes represent diffraction limited objects, they were detected as 3D particles and the positions of their center of mass were calculated. Finally, co-localization was assumed when the distance between the centers of the mass of two neighboring particles was below 300 nm, the resolution limit of the microscope.

### Theoretical calculation of the concentration-dependent quenching of FlAsH dyes inside viral particles

To calculate the theoretical fluorescence intensity of HIV-1 pseudoviral particles containing a given proportion of TC-tagged NCp7 molecules relative to the fluorescence intensity of HIV-1 pseudoviral particles containing 100% TC-tagged NCp7 molecules, we modeled the system of tagged NCp7 proteins in the viral core as a network with space-separated dyes as described in^31^. In this network, the protein-bound dyes are assumed to be hard spheres with the dye being a point emitter in the center of the sphere. All spheres are assumed to be localized in the volume *V* of the viral core. In reality, NCp7-TC/FlAsH has the dye bound on its surface instead of its center, but as NC binds to RNA only in a “head-to-tail” configuration^6^, this still positions the dyes one NC diameter apart.

A nearest-neighbor dye-dye distance distribution *H*_*χc*_*(r)*^62^ can be calculated from the quantity of dyes contained in the volume V and the percentage of tagged NCp7 proteins *χ*. We assume that each excited dye can either (i) emit light with a quantum yield *QY*, (we assume that k_r_ + k_nr_ = 1 a.u., in these units k_r_ equals numerically QY), (ii) non-radiatively transfer energy through homo-FRET to one of its nearest-neighbor dyes (at a rate *k*_*ET*_), or (iii) dissipate energy non-radiatively due to packing -induced quenching related to the close proximity of the dyes (at a rate given by *εk*_*ET*_), where *ε* is the packing-induced quenching factor. As a result, the effective quantum yield *η(r)* of the dyes having a nearest-neighbor distance of *r* corresponds to:

1 $$\eta ({\rm{r}})={\rm{QY}}\frac{1}{1+\varepsilon {k}_{ET}}={\rm{QY}}\frac{{r}^{6}}{{r}^{6}+{R}_{Q}^{6}}$$

where *R*_*Q*_ = *N*^*1/6*^*ε*^*1/6*^*R*_*F*_ is the effective quenching radius, with *R*_*F*_ being the Förster radius for homo-FRET and *N*, the effective number of nearest neighbor dyes. *N* is dependent on *r*, calculated as described in^62^.

Integrating (1) over the full dye-dye distance distribution gives the total effective intensity of the dye ensemble:

2 $${I}_{\chi c}=\chi {c}_{NCp7}\mathop{\int }\limits_{r}\,{H}_{\chi c}(r){\eta }_{r}dr$$

Note that in real experiments *I*_*χc*_ is also multiplied by normalizing factors that depend on multiple instrumental parameters. However, if the experiments are performed in the same conditions, the relative intensity between two viral particles will depend only on the quantity of tagged NCp7 proteins in the viral core.

The following assumptions were made for the calculation of the relative fluorescence intensities: (a) FlAsH binds to all TC tags present in the pseudovirus; (b) NCp7-TC and NCp7 are assumed to be spheres with a diameter of 2.2 nm (PDB ID: 1ESK, 1BJ6, 2EXF, 2L4L); (c) an intact viral particle contains 2250 NCp7 molecules^4^; (d) the FlAsH-TC/NCp7 quantum yield *QY* is taken as 49%^17^; (e) *R*_*f*_, the homo-FRET Förster radius, was calculated as 4.46 nm based on spectra of TC-bound FlAsH in water^17^, which are assumed to not shift as a result of packing-induced quenching.

The unknown parameters for the system are V and ε. The V parameter depends on how tightly bound is the NCp7-RNA complex, so it has a lower limit of 18817 nm^3^ (total volume of two copies of viral RNA + 2250 NCp7 molecules) and an upper limit of 206000 nm^3^ (volume of the viral core approximated as a truncated cone with a narrow end radius of 15 nm and a broad end radius of 30 nm and 125 nm height)^29^. Based on a set of intensity ratios of intact viral particles containing various percentages of NCp7-TC relative to virions containing 100% NCp7-TC, the two unknown parameters were extracted by numerical nonlinear fitting (using a Nelder-Mead algorithm). The calculations were repeated more than 20 times starting from different initial parameters in order to ensure that the global minimum was found. Calculations were performed with Wolfram Mathematica 10.0.

The datasets generated and/or analyzed during the current study are available from the corresponding author on reasonable request.

## Supplementary information

Supplementary Information
