# Supplementary material for: Quantitative monitoring of the cytoplasmic release of NCp7 proteins from individual HIV-1 viral cores during the early steps of infection
Source: Sci Rep. 2019 Jan 30;9:945. doi: 10.1038/s41598-018-37150-0 (PMC6353972; doi:10.1038/s41598-018-37150-0)
Supplement: Supplementary file 1 — Supplementary Information [file 41598_2018_37150_MOESM1_ESM.pdf]

Quantitative monitoring of the cytoplasmic release of NCp7 proteins from individual HIV-1 viral cores during the early steps of infection

Sarwat Zgheib‡, Iryna Lysova‡, Eleonore Réal, Oleksii Dukhno, Romain Vauchelles, Manuel Pires, Halina Anton, Yves Mély**

SUPPLEMENTARY MATERIAL:

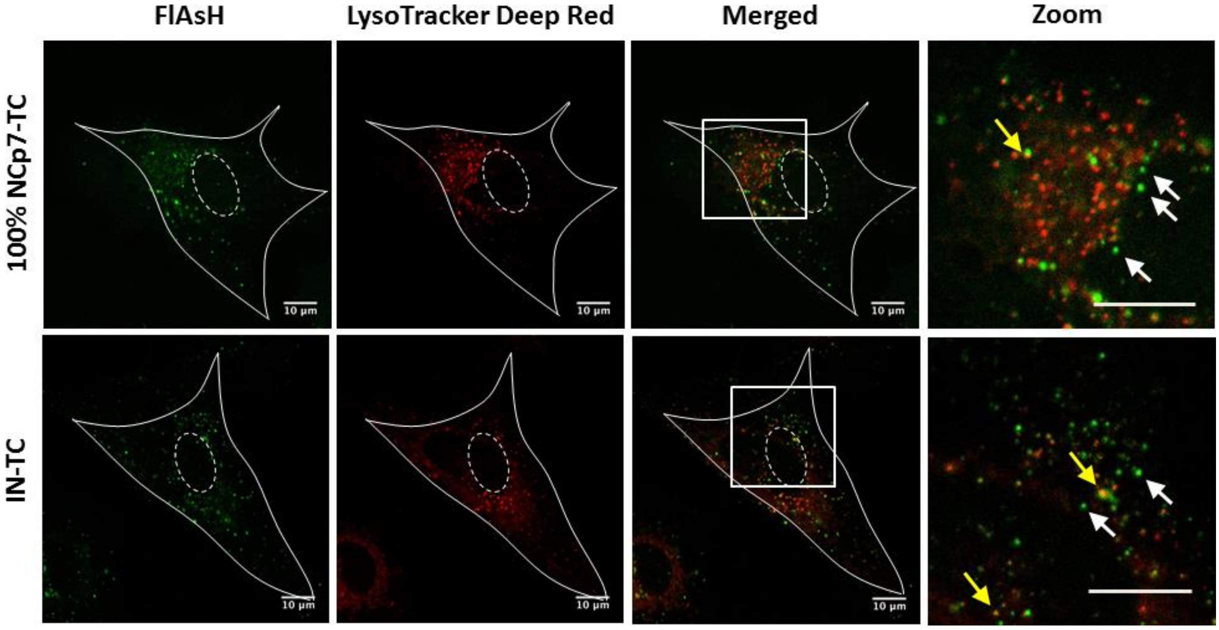

Figure S1: Co-Localization of FlAsH-labeled HIV-1 pseudoviruses containing 100% NCp7-TC or IN-TC with the lysosomal staining.

Typical confocal images of FlAsH-labeled pseudoviruses and LysoTracker Deep red labeled lysosomes in HeLA cells infected during 2 hours. White and yellow arrows in the zoomed view represent free and lysosome-trapped pseudoviruses, respectively. The co-localization analysis performed in the whole 3D stack using the JACoP plugin for ImageJ [63] revealed that only 14±8% of the FlAsH fluorescent spots co-localize with the spots in the red channel (N=20).

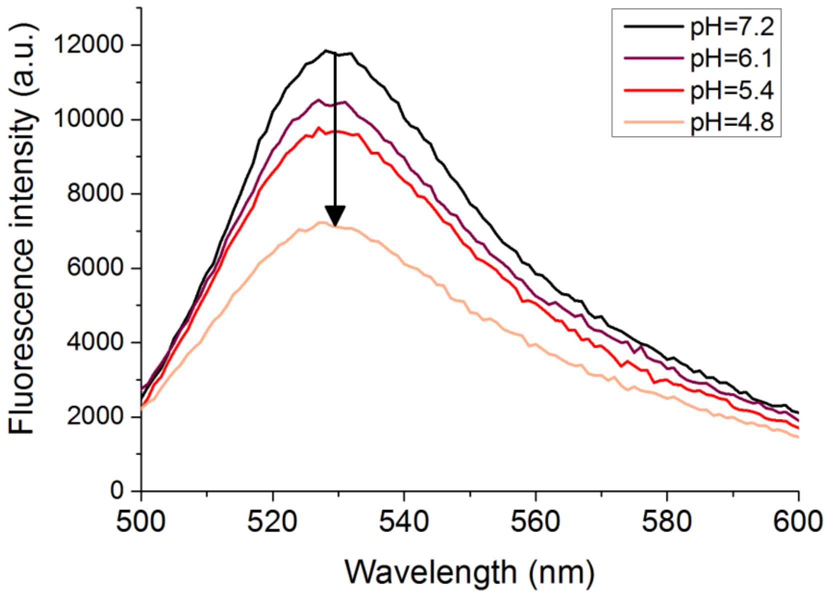

Figure S2: Fluorescence emission spectra of FlAsH-labeled HIV-1 pseudoviruses containing 15% NCp7-TC measured at different pH values (λ_{ex} =480 nm).
